# Characterizing Deep Brain Stimulation effects in computationally efficient neural network models

**DOI:** 10.1186/1753-4631-5-2

**Published:** 2011-04-15

**Authors:** Alberta Latteri, Paolo Arena, Paolo Mazzone

**Affiliations:** 1 DIEEI - Università di Catania, v.le A. Doria 6, Catania, Italy; 2 C.T.O. "A. Alesini" via S. Nemesio, 21 - 00145 Roma, Italy

## Abstract

**Background:**

Recent studies on the medical treatment of Parkinson's disease (PD) led to the introduction of the so called Deep Brain Stimulation (DBS) technique. This particular therapy allows to contrast actively the pathological activity of various Deep Brain structures, responsible for the well known PD symptoms. This technique, frequently joined to dopaminergic drugs administration, replaces the surgical interventions implemented to contrast the activity of specific brain nuclei, called Basal Ganglia (BG). This clinical protocol gave the possibility to analyse and inspect signals measured from the electrodes implanted into the deep brain regions. The analysis of these signals led to the possibility to study the PD as a specific case of dynamical synchronization in biological neural networks, with the advantage to apply the theoretical analysis developed in such scientific field to find efficient treatments to face with this important disease. Experimental results in fact show that the PD neurological diseases are characterized by a pathological signal synchronization in BG. Parkinsonian tremor, for example, is ascribed to be caused by neuron populations of the Thalamic and Striatal structures that undergo an abnormal synchronization. On the contrary, in normal conditions, the activity of the same neuron populations do not appear to be correlated and synchronized.

**Results:**

To study in details the effect of the stimulation signal on a pathological neural medium, efficient models of these neural structures were built, which are able to show, without any external input, the intrinsic properties of a pathological neural tissue, mimicking the BG synchronized dynamics.

We start considering a model already introduced in the literature to investigate the effects of electrical stimulation on pathologically synchronized clusters of neurons. This model used Morris Lecar type neurons. This neuron model, although having a high level of biological plausibility, requires a large computational effort to simulate large scale networks. For this reason we considered a reduced order model, the Izhikevich one, which is computationally much lighter. The comparison between neural lattices built using both neuron models provided comparable results, both without traditional stimulation and in presence of all the stimulation protocols. This was a first result toward the study and simulation of the large scale neural networks involved in pathological dynamics.

Using the reduced order model an inspection on the activity of two neural lattices was also carried out at the aim to analyze how the stimulation in one area could affect the dynamics in another area, like the usual medical treatment protocols require.

The study of population dynamics that was carried out allowed us to investigate, through simulations, the positive effects of the stimulation signals in terms of desynchronization of the neural dynamics.

**Conclusions:**

The results obtained constitute a significant added value to the analysis of synchronization and desynchronization effects due to neural stimulation. This work gives the opportunity to more efficiently study the effect of stimulation in large scale yet computationally efficient neural networks. Results were compared both with the other mathematical models, using Morris Lecar and Izhikevich neurons, and with simulated Local Field Potentials (LFP).

## Background

PD is a degenerative disorder of the central nervous system that often impairs motor skills, speech, and other functions[1]. It is characterized by muscle rigidity, tremor, a slowing of physical movements (bradykinesia) and, in extreme cases, a complete loss of physical movement (akinesia). The primary symptoms are the results of decreased stimulation of the motor cortex by the basal ganglia and other brain stem structures, traditionally considered as a consequence of the insufficient formation and action of dopamine, which is produced in the dopaminergic neurons of the Substantia Nigra reticulata (SNr). Other symptoms may include high level cognitive dysfunction and subtle language problems, postural instability and gait disturbances. In some cases, it would be inaccurate to say that the cause is "unknown", because a small proportion is caused by genetic mutations. It is possible for a patient to be initially diagnosed with PD but then to develop additional features, requiring revision of the diagnosis[2].

At present, there is no cure for PD, but medications or surgery can provide relief from the symptoms. The most widely used form of treatment is L-dopa in various forms. However, only 1-5% of L-DOPA enters the dopaminergic neurons. The remaining L-DOPA is often metabolized to dopamine elsewhere, causing a wide variety of side effects. Due to feedback inhibition, L-dopa results in a reduction in the endogenous formation of L-dopa, and so eventually becomes counterproductive[3]. In the 1990s the surgical ablation has been used to treat PD. Ablative brain surgery is the surgical lesion by burning or freezing or with chemical substances of brain tissue to treat neurological or psychological disorders. The thalamus was a potential target for treating tremor, especially in the Ventral Intermediate Nucleus (VIM) and Centrum Medianum-Parafascicular (CM-Pf) nuclei. The lesions caused by this type of surgery are irreversible, so generally DBS surgery is considered preferable to lesion because it has the same effect and is adjustable and reversible [4-6]. Treating PD with surgery was once a common practice, but after the discovery of levodopa, surgery was restricted to only a few cases. DBS is a surgical treatment involving the implantation of a medical device called a brain pacemaker, which sends electrical impulses to specific parts of the brain. DBS in selected brain regions has provided remarkable therapeutic benefits for otherwise treatment-resistant movements and affective disorders such as chronic pain, PD or essential tremor and dystonia[7]. Despite the long history of DBS, [8], its underlying principles and mechanisms are still unclear. DBS directly changes brain activity in a controlled manner, its effects are reversible (unlike those of lesioning techniques). The Food and Drug Administration (FDA) approved DBS as a treatment for essential tremor in 1997, for PD in 2002 [9], and dystonia in 2003 [10]. DBS leads are placed in the brain according to the type of symptoms to be addressed. For dystonia and symptoms associated with PD (rigidity, bradykinesia/akinesia and tremor), the lead may be placed in either the Globus Pallidus or Subthalamic Nucleus [11]. The right side of the brain is stimulated to address symptoms on the left side of the body and vice versa. DBS does not cure PD, but it can help to manage some of its symptoms and subsequently to improve the patient's quality of life [12]. Presently, the procedure is used only for patients whose symptoms cannot be adequately controlled with medications, or whose medications have severe side effects [13]. Its direct effect on the physiology of brain cells and/or neurotransmitters is currently debated, but it is apparent that sending high frequency electrical impulses into specific areas of the brain can mitigate symptoms [14] and/or directly decrease the side effects induced by Parkinsonian medications, [15], since it allows a sometimes huge decrease in medications, making the medication regime more tolerable. More recent neurophysiological data suggest that the DBS can modify also the connections among the cellular networks, giving rise to a holistic interpretation of DBS action these aspects that should be also considered in the future [16].

There are a few sites in the brain that can be targeted to achieve different results, so each patient must be assessed individually, and the particular site (or concurrent sites) to be stimulated is chosen based on the particular needs. Traditionally, the two most common sites are the Subthalamic Nucleus (STN) and the Globus Pallidus internus (GPi), but other sites, such as the CM-Pf [17] and Nucleus Tegmenti Peduncolopontini (PPTg) have recently shown important benefits for PD treatment [18]. A tailored DBS with the targeting of multiple nuclei was proposed to obtain the high clinical results in complex parkinsonian syndromes [18]. The main objective of stimulation is to re-establish desynchronization via a pulse train, whose parameters are selected by the Neurosurgeon at the main aim to decrease the disease symptoms. The patient with PD has a high synchronization into band β (10÷30 Hz) of the activities STN and GPi (brakinesia., akinesia., etc.), whereas the patient with L-Dopa into same band has high desynchronization, with an improvement of movements [19]. These results confirm what is expected from the Gate Control Theory [20], which states that the synchronization of neuronal activity obstructs information flow in brain structures, whereas, the desynchronization allows information flow.

A increase of the neuronal synchronization causes a increase of power spectral density (PSD) of the Local Field Potential (LFP) taken from STN and GPi, whereas, the neuronal desynchronization causes a decrease of the PSD.

Such a desynchronization in the β band takes place also when the patient is subjected to DBS, sending a pulse train with frequency larger than 70 Hz [21]. The DBS of the STN and GPi, involves a synchronization of neurons in the high frequencies and a desychronization in the β band. These stimuli have the common goal of reducing the synchronized activity of specific clusters of neurons, in order to desynchronize the target population, re-establishing a normal physiological activity in a highly synchronized population.

The first step toward the understanding of the dynamic effect of the DBS on the brain nuclei, is to design a model of these areas and simulate the effects caused by the stimulation. At this aim, after a deep scanning of the state of the art, in [22], a model of the neuronal BG was derived using a neural network made up of Morris-Lecar neurons, arranged in a specific population and showing, without any external input, basic oscillations mimicking the Parkinsonian tremor. As in [22], the positive effect of an electrical stimulation induced by implanted electrodes was demonstrated in such a population model. Therefore this model was initially taken into consideration as a reference. Then a fundamental issue, when the need of simulating large scale populations arises, lies in the fact that the number of floating point operations needed to simulate a time unit for a single neuron is relevant for the time needed to appreciate the results of the whole population. This led us to analyze other neuron models that could reveal the same characteristics (basically autonomous bursting oscillations) as the Morris Lecar model, but being at the same time much less demanding as regards the computational burden. Therefore, after analysing, in the first part of this paper in the method section, the effect produced by a population of Morris Lecar units, the same conditions were reproduced using a population consisting of Izhikevich neurons [23], where parameters were selected so as to show a resonant-like bursting characteristics, similar to the Morris Lecar model, but with a much less computational power demand. Of course, all the other characteristics, like the synaptic dynamics and all the other relevant parameters, were left unchanged.

In the second part of this paper, this new reduced order model gave us the possibility to increase, where needed, the population size using roughly the same computation time. The positioning of the electrodes within the population so built revealed the expected effects i.e. a desynchronization within the neurons, which led to a spreading of the power spectral density over a wide frequency range. This effect even increases in the presence of multiple stimulating sites within the population.

In this paper the population targets simulated represented the (STN) and Globus Pallidus Externus (GPe) but recent researches have a new target like the PPn [16].

## Methods

The patients series and selection criteria, surgical planning and stereotactic technique are described elsewhere[18]. In this paper, as also reported above, attention is focused on the reduced order mathematical models of neural structures able to show pathological PD conditions and to show the positive effect of electrical stimulation. The first mathematical model that we used mimics some important characteristics of the dynamical behaviour of a population of neurons of the STN, e.g. a burst and spike activity. In particular, our model displays oscillatory activity, as observed in experimental investigations of brain areas, such as the basal ganglia and the STN, relevant for the characteristics for PD [24]. In this method we used the well known Morris-Lecar equation as a spike generator [25]. The single STN neuron was described by a set of four time-delayed differential equations, whose parameters and their role in the overall neuron/connection dynamics are described below:(1)(2)(3)(4)

where *v_j _*denoted the membrane potential of the *j_th _*neuron and *w_j _*was an auxiliary variable Eq. 4 describes the evolution of the parameter  (the index *s *denotes synaptic), relevant for the synaptic coupling and modulating the dynamics of the current  (see Eq. 10). The  is calculated as a function of the membrane potential *v_j_*. The other parameters denote:

*C*: membrane capacitance;

*g_ca_, g_k, _g_l_*: leak, Ca++, and K+ conductances through membranes channel;

*v_ca_, v_k, _v_l _*: equilibrium potential of relevant ion channels;

*v_1_, v_2, _v_3, _v_4,_*: tuning parameters for steady state and time constant.

The dynamics of the calcium and potassium ion channels, responsible for the neurons spiking were controlled by a set of three equations:(5)(6)(7)

The neuron model is dimensionless, and we modulated the parameters to obtain realistic bursting patterns [26,27].

The dynamics of the neuron was controlled by external currents: a slowly varying current , whose dynamics is described in Eq. 3; this current had been proposed by Rinzel and Ermentrout [28] as a source of bursting, which reflects the inhibitory feedback from the GPe.

The GPe neurons were excited by the STN activity *v_j_*, and after a time delay τ*_j _*the activity in the GPe, in turn, led to an inhibition of the STN neurons mediated by recurrent pathways [29-33]. The bursting behaviour and its frequency were controlled, via Eq. 3 by the Gaussian distributed parameters *ε_j _*and by the parameter α. The former induces a slightly different natural frequency within each neuron, whereas the latter modulates the inhibitory response of the GPe neurons. Background activity introduced by external and internal sources was modelled by a spatially incoherent exponentially correlated noise source with amplitude *D_noise_*[34]. The exponentially correlated noise was calculated by using a second order algorithm with decay time τ*_noise_*. These two last parameters contribute to the definition of . The  was described by a set of two time-delayed differential equations:(8)(9)

where ξ(t) is Gaussian distributed, zero mean noise with unit standard deviation [34]. The neuronal dynamics displayed by the simplified model STN neurons are characterized by a bursting activity, wherein the simulations the bursting frequency is chosen to be close to frequencies usually observed in patients suffering from parkinsonian tremor. The bursts are formed by a small number of spikes, six to ten per burst, which are controlled by the feedback from the GPe.

The bursting pattern displayed by the network elements is qualitatively similar to the bursting pattern observed in wide areas of the nervous system [35,36,26,27]. For illustration, we represent the neurons in the populations as arranged in square lattices. Two stimulation protocols were used: the first one using one electrode positioned at the lattice center, and a second one consisting of four stimulation electrodes, equally spaced within the population.

For the numerical integration of the system we used the routine by Runge-Kutta method of order four.

Simplifying the complex organization of the neuronal population of the STN, we mimic excitatory couplings between different neurons of the population. In literature, the origin of the synchronized activity established between neurons in pathological conditions is not known, but could be induced by the modification of the bursting activity of the neurons.

Also, in our model, single spikes are not able to cause a significant change of the spiking times of the other neurons, only synchronized bursts are able to induce such a change in the firing pattern.

To design the connection within STN neurons we used a simple excitatory coupling () that does not take into account any input from other BG nuclei. The synaptic interaction is modelled as suggested by Terman [37,38].

The action potential results in an opening of the corresponding ion gates, causing the income of the  current (Eq. 10). This is composed of the local gating variables , weighted with a distance-dependent function and multiplied with a maximal gating term and the potential difference corresponding to the glutamatergic synapses present in the STN [38-40]:(10)

**Figure 1:**
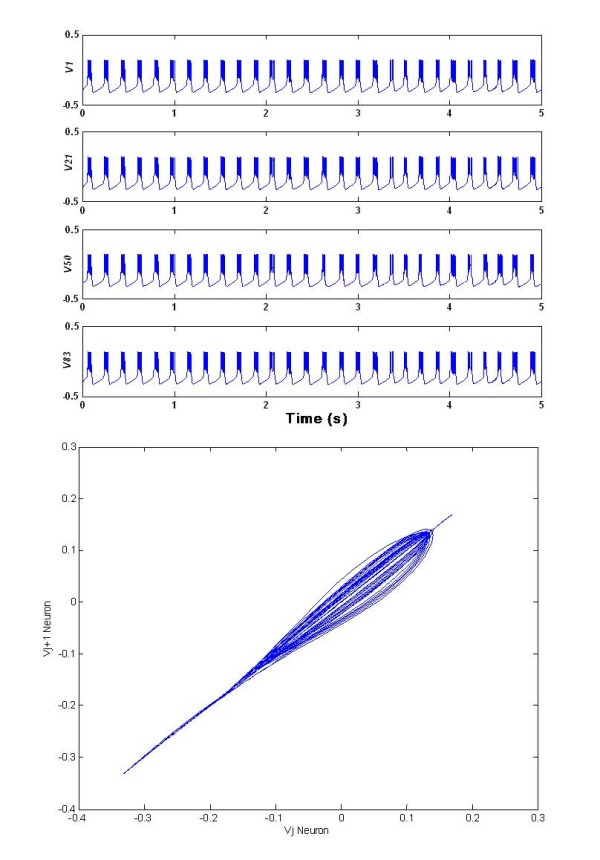
**Synchronized activity of the coupled neuron population**. The membrane potentials of four neurons: *v_1_(t) v _21_(t) v_50_(t) v_83 _(t)*, randomly selected within a regular lattice of 100 neurons. Subscripts indicate their position in the lattice, ordered lexicographically. The membrane potential dynamics show the characteristic synchronized bursting activity, whereas in Figure 1, bottom side, the plot of the membrane potential on two different randomly selected neurons within the network is depicted, outlining the large amount of synchronization. The upper part of the diagonal shows a small side dispersion, due to the slightly different times of the bursting activity. Typical numerical values for the different variables are as follows: *v_j_(t)*∈ [-0.3,0.14]; *I_jslow _*(*t*) ∈ [-0.008,0.012; . Parameters: *g_ca_*= 1.0; *g_k_= *2.0;*g_l _*= 0.5;*v_ca_*= 1.0; *v _k_*= - 0.7; *v_l_*= -0.5; *v_1 _*= -0.01; *v_2 _*= 0.15; *v_3 _*= 0.1; *v_4 _*= 0.145; *C*= 1.0; *φ *= 1.15; v* = -0.22; *α *= 0.0; *τ_j _*= 10; Gaussian distributed *ε_j _*= 2·10-3(± 2·10-5) mean (± standard deviation); *α_s _*= 0.1; *β_s _*= 0.05;*θ_s _*= 0.2; *σ_s_*= 0.02; *s*= 0.4; *σ*_*g *_= 0.5; *v*_*s *_= -0.85; N = 100; *dl *= 0.1; *D*_*noise *_= 0.00001; *τ*_*noise *_= 5; *c*_*s *_= 0.1; *c*_*n*_= 2.0584.

Here ||*y_j _*- *y^k^*|| is the distance between *k_th _*the *j_th _*and the neuron. The details of connections within the populations on which most of the current studies of PD focus (the BG and especially the STN) are poorly understood [37,40,41,38]. However, from the hippocampus and the visual cortex, we know that local rather than global connections are developed [42,43]. Hence, we used a local pattern of synaptic interaction.cn is a normalization factor. N is the number of neurons within the population. The neurons were arranged on a square lattice (lattice distance dl), as shown in Figure 1.

The stimulation was applied via one or four electrodes located within the network. The strength of the stimulation typically decays with distance from the stimulation electrode. Which compartments of a neuron are activated by an electrical extracellular stimulation is still not clear [44,45]. The final result of the stimulation is most probably a combination of excitatory action directly at the soma of the neuron together with an activation of afferent fibres [46,31]. Therefore in our mathematical model we elaborated excitatory actions, which take place at the soma and within the population mediated by excitatory synapses connecting the STN neurons [47,40,30,41,48], and inhibitory actions coming from an activation of inhibitory afferent fibres [30-33].

Hence, the absolute value of the stimulation current was used to control the slowly varying current. Therefore, this type of stimulation mimicked the activation of afferent inhibitory fibres. The exact interplay between these different types of action remains unknown, to date, and is essential in determining the shape of the electrical pulses [49]. To cope with this challenge we first investigated excitatory effects. The corresponding stimulation term was given in Eq. (1) as . Here *s*_1 _= 1 is used to weight the local effectiveness of the stimulation and the step function *X*_1 _defines the onset and offset of the excitatory stimulation acting directly at the soma of the stimulated neuron.

**Figure 2:**
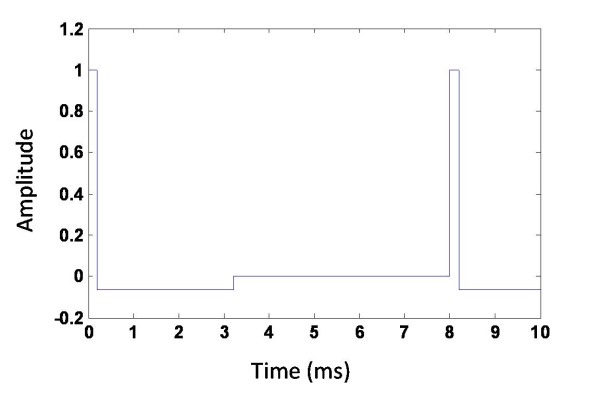
**High-frequency pulse train W(t) delivered through the electrode**. Pulses were permanently supplied at a frequency of 130 Hz. The figure shows the typical shape of a charge balanced pulse.

For the standard high-frequency DBS we used biphasic stimulation pulses. The first short positive pulse of length 0.2 ms was followed by a longer negative pulse of length 3.0 ms. The amplitude of the second pulse was adjusted such that the biphasic pulse was charged balanced, e.g. the total amount of applied electric charge was zero (Figure 2).

Biphasic pulses are used in clinical applications to avoid a charge deposition in the tissue. The stimulation was administered through one of four electrodes positioned in the center of the population. The spatial activation profile of the stimulation is not known in detail [50]. For simplicity, we assumed a certain level of homogeneity in the network and supposed that the stimulation signal uniformly and exponentially decays with increasing distance to the stimulation electrode. The stimulation current was given by:(11)

where ||*y_j _*- *y^k^*|| is the distance between the *k_th _*electrode and *j_th _*neuron, and *c*_s _is the parameter that controls the strength of the stimulation. *x*_1_(*t*) (Eq. 1) determines the onset and offset of the excitatory stimulation. *W*(*t*) is the continuous pulse train consisting of a high-frequency repetition of the biphasic pulses presented through the electrode (Figure 2). *c_n _*is a normalization factor, which is dependent on the number of stimulation electrodes used and which guarantees that the total amount of stimulation remains independent on the number of electrodes used.

To analyze the synchronization of dynamics of the neuronal population, we quantified the phase of the network considering the distribution of the phases. In the network the busting activity was the prominent dynamics, so we identified the bursting onset as a trigger to detect the phase displacement, calculated, for a single neuron j, as follows:(12)

where *t *∈ [*t*_*k*, _*t*_*k*+1_], and *t*_*k *_was the onset time of the *k*_*th *_burst of the neuron. The quantity characterizing the synchronization activity in oscillatory networks is given by:(13)

Here R is the measure of synchronization and Θ is mean phase. It results that 0 ≤ *R*(*t*) ≤ 1 for all times t; R = 1 corresponds to perfect in-phase synchronization, whereas R = 0 means complete desynchronization [5].

**Figure 3:**
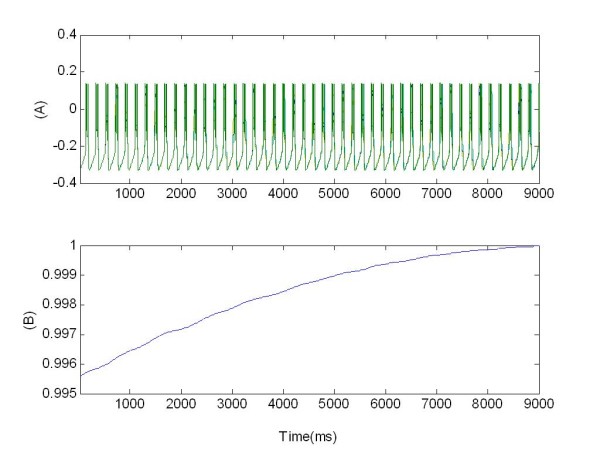
**Synchronized activity of the coupled population of 100 neurons**. (A) Bursting pattern of membrane potentials *v_j_(t)*; (B) synchronization measure R given by Eq. 13.

Therefore with the synchronization measure R we were able to reliably detect in-phase synchronization and desynchronization. As an example, in Figure 3 the value of R(t) is reported for the whole network of 100 neurons already discussed (see Figure 1). Due to the random initial conditions, R starts from an initial (already high) value and reaches soon its maximum.

Stimulation consists in introducing a stimulation current that influences Eq. 1. In this equation we remind that the parameter *X*_1 _controls the onset and offset of the excitatory stimulation acting directly at the soma of the stimulated neuron. Moreover W(t) is a pulse train with a fixed high-frequency equal to 130 Hz, consisting of a repetition of the biphasic pulses delivered through the electrode. This approach was applied in both cases studied, i.e. when only one electrode is implanted at the centre of the network and when four electrodes are positioned symmetrically with respect to the lattice. Multi target stimulation reflects the common neurosurgical protocol. However, both the techniques (single and multi-electrodes) have the common goal of reducing the synchronized activity of the target population, either by re-establishing a normal physiological activity in a highly synchronized population of neurons or by reversibly mimicking a tissue lesioning (DBS).

### Stimulation methods via one central electrode

**Figure 4:**
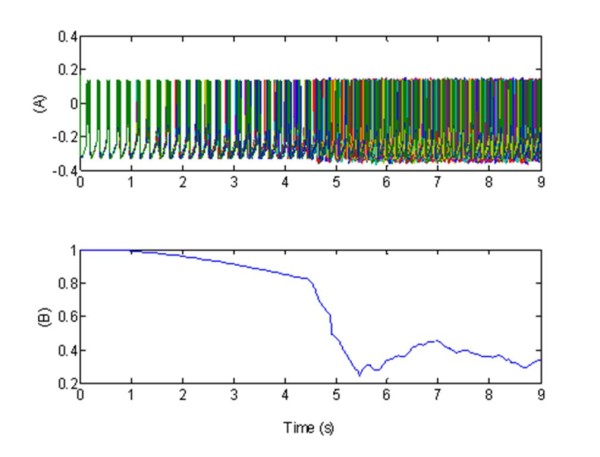
**Effect of a simulation of the standard high-frequency deep brain stimulation, via one central electrode, in terms synchronized activity of the coupled population of 100 neurons**. (A) Bursting pattern during the excitatory stimulation of all the membrane potentials *v_j_(t)*. These show the characteristic synchronized (before stimulation) and desynchronized (after stimulation) bursting activity; (B) Synchronization measure R given by Eq. 13. In plots (A-B), the stimulation signal W(t), (see Figure 2), was formed by a 130 Hz pulse train composed of biphasic pulses (0.2 ms positive followed by 3 ms negative stimulation). The stimulation was supplied via one electrode situated in the central part of the network. Parameters: *X_1_*= 1 for t ∈ [4.5, 9]s, excitatory stimulation; *X_1 _*= 0, elsewhere,

Figure 4 reports a simulation with 100 neurons organized in regular network with one central stimulating electrode.

The figure shows that, in absence of stimulation, the neurons bursting was strongly synchronized in-phase. At *t *= 4.5 _s _we introduced the stimulating signal: this caused a desynchronization for *t *_= _4.5 _s _, leading a large decrease in R(t) (average value R(t) = 0.4). The pulse input, simulating DBS, acted so as to mask the synchronizing effects of the excitatory interconnections within the STN.

### Stimulation methods via four electrodes

in the multi electrodes case, the stimulation strength (in terms of current in Eq. 11) decayed with distance from the stimulation site and each neuron received a mixture of the four stimulation signals.

**Figure 5:**
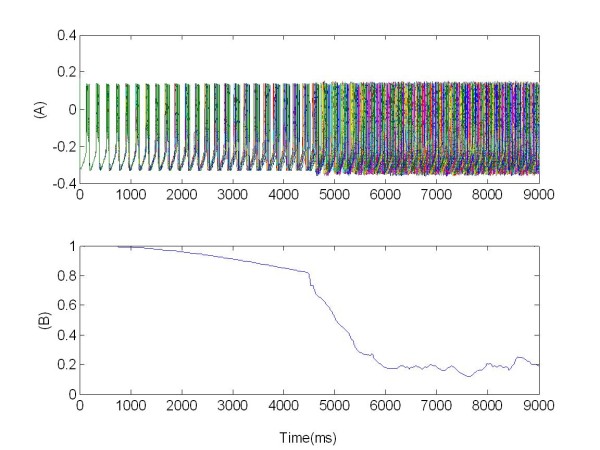
**Effect of a simulation of the standard high-frequency deep brain stimulation, via four central electrodes, in terms synchronized activity of the coupled population of 100 neurons**. (A) Bursting pattern during the excitatory stimulation; the membrane potentials *v_j_(t) *of 100 neurons are plotted, showing the characteristic synchronized and desynchronized bursting activity; (B) Synchronization measure R given by Eq. 13. In plots (A-B), the stimulation signal W(t), Figure 2, was formed by a 130 Hz pulse train composed out of biphasic pulses (0.2 ms positive followed by 3 ms negative stimulation). The stimulation was supplied via four electrodes situated in the central network. Parameters: *X_1 _*= 1 for t ∈ [4.5,9]s, excitatory stimulation; *X_1_*= 0 elsewhere. DBS: Standard high-frequency deep brain stimulation.

The DBS with four electrodes resulted in a good desynchronization for excitatory stimulation, indicated by a vanishing synchronization measure R (Figure 5(B)). The mean synchronization measure was R(t) = 0.15 for excitatory stimulation whereas, without stimulation, the index of synchronization was R(t) = 0.9.

## Results

The model of the neural network that we used to simulate the biological behavior, in the previous section, was made up of a limited number of neurons, imposed by the limitations of computational resources.

Till now we modelled the neural network using Morris Lecar neurons consisting of six differential equations: in these conditions, considering a reasonable computation burden, we could analyze a number of neurons not larger than 100. For this reason we decided to study a reduced order neuron model, with less differential equations but preserving the same characteristics.

We used the Izhikevich model equation, in particular the chattering model for bursting neurons [23]. This model offers a reduced complexity, if compared with the Morris Lecar equations, retaining, at the same time, the most important features. In particular the neurons can fire with stereotypical bursts of closely spaced spikes [51].

All the other characteristics, like the synaptic dynamics (*I_jsyn_*), the noise component (*I_jnoise_*), the stimulation current (*I_jstim_*) and other relevant parameters for the network, were left unchanged. The Izhikevich parameters were suitably modified in order to allow a (time) comparison with the Morris Lecar model.

The single Izhikevich membrane neuron model was described by the two differential equations (14 and 15). In dimensionless form the dynamics of the membrane potential *v_j _*of the *j_th _*neuron, including the dynamics of the synaptic coupling, taken from Eq.(4), is described by the following set of equations:(14)(15)(16)

Here *u_j _*is an auxiliary variable. The term was obtained by fitting the spike initiation dynamics of cortical neurons so that units of *v_j _*correspond to mV and units of time correspond to ms. We modulated the parameters so as to obtain realistic bursting patterns. The parameter *a *determines the rate of recovery, whereas *b *determines the sensitivity of the recovery variable *u_j _*to the membrane potential *v_j _*.

The parameter *c *determines the after-spike reset value, which depends on fast high threshold conductance. Similarly, the parameter *d *determines the after-spike reset of the recovery variable, which depends on slow high-threshold conductance.

The Eq. 16, was the same as Eq.4 related the system studied in the previous section. The *I_jnoise _*was calculated offline by using a second order algorithm with decay time τ*_noise_*[34]. We used a numerical integration method based on the Eulero algorithm.

**Figure 6:**
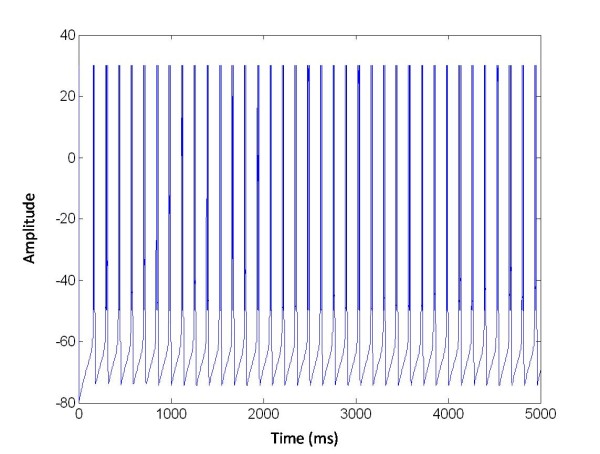
**Activity of one neuron described by Izhikevich equations**. The membrane potentials *v(t) *of one neuron shows the characteristic spiking and bursting activity. Typical numerical values for the different variables are as follows. Parameters: *D_noise _*= 0.00001. *n *= 0.001; , *a *= 0.02, *b *= 0.2;*c *= 50, *d *= 0.7, *T_1 _*= 22, *α_s _*= 0.1; *β_s _*= 0.05; *θ_s _*= 0.2; *σ_s _*= 0.02;

The parameters  and *d *control the bursting behaviour and its frequency. Also in this case the spikes that form one burst are from 6 to 10 per burst. The dynamics of a single neuron described by the model presented is depicted in Figure 6.

To shape the connections we used the same synaptic current seen in the method section to connect Morris Lecar neurons, except that we introduced a Gaussian distribution into the distance among neurons to better emulate the realistic case.(17)

Here ||*y_j _*- *y_k_*|| is the distance between the *k_th_*and the *j_th _*neuron and *G_r _*is a random variable with Gaussian distribution, zero mean and 0.1 standard deviation; it is added to the distance among neurons.

**Figure 7:**
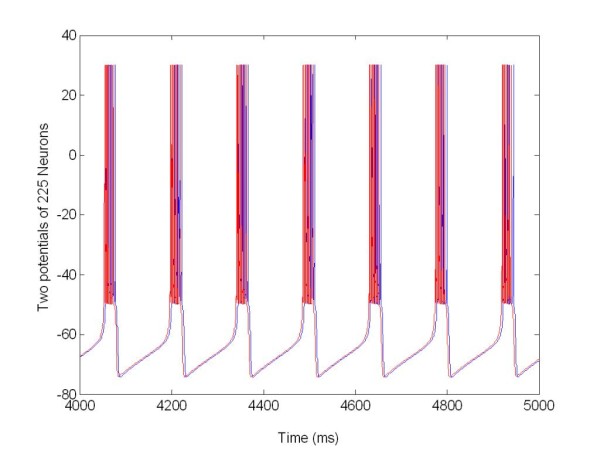
**Synchronized activity of the coupled population of neurons**. The membrane potentials *v_j_(t) *of 2 on 225 selected neurons, in one second, show the characteristic synchronized bursting activity. Parameters: *T_1 _*= 22, *α_s _*= 0.1; *β_s _*= 0.05; *θ_s _*= 0.2; *σ_s _*= 0.02;*g^¯^_s _*= 0.2;*v_k _*= -0.8; *c_n_^c ^*= 2.1765;

N is the number of neurons in the network organized in this random lattice (see Figure 7).

After the definition of the reduced neuron model and the corresponding network, we use the same stimulation protocol as that applied with the Morris Lecar system but with a different number of neurons.

Using numerical methods we investigated the dynamical properties of a neural population consisting of 250 neurons. In the simulations we used the same periodic train pulse discussed above. In the first case, as seen before, we stimulated with an electrode in the middle of the network and then with four electrodes positioned in the centre of the population. A further step was to connect two identical populations to appreciate the effect of the stimulation in both populations, resembling the effect of multiple nuclei, typical of DBS.

**Figure 8:**
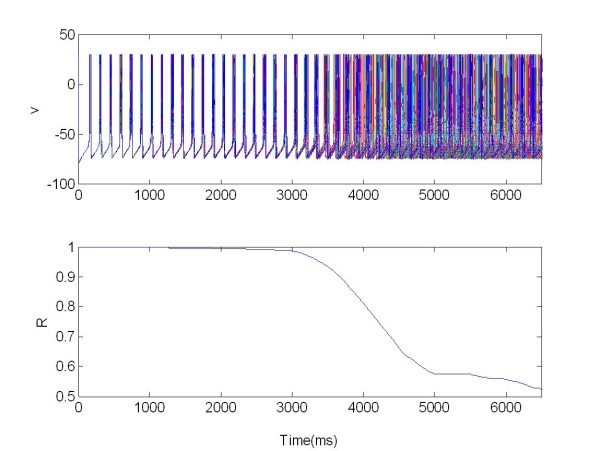
**The membrane potentials *v(t) *of one population of 225 neurons organized in a random network and stimulated via one electrode positioned in the middle of the network**. X = 1 for t ∈ [3,6.5]s, excitatory stimulation; X = 0 elsewhere.

Also in this case the R parameter was used as the index of synchronization (Eq. 13). In this section we study the synchronization and desynchronization with the same protocol as in method section. So in the first equation with the parameter X we can determine the onset and offset of the excitatory stimulation, with *s *= 50 and W(t) as explained above. In Figure 8 a population of 225 Neurons organized in random network was stimulated with a central electrode.

This shows that the neuron bursting was strongly synchronized in-phase and R(t) = 1 for t ∈ 0[3]s. The mean synchronization measure was R(t) = 0.6 for *t *∈ [3,6.5]s. In this case the network desynchronized after applying the stimulation signal.

**Figure 9:**
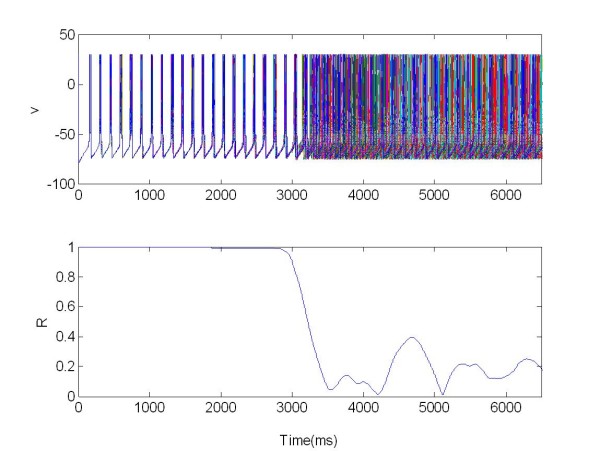
**The membrane potentials *v(t) *of 225 neurons are plotted: they show the characteristic synchronized and desynchronized bursting activity**. The population was organized in random network and stimulated via four electrodes. R: Synchronization measure X = 1 for t ∈ [3,6.5]s, excitatory stimulation; X = 0 elsewhere.

The DBS with four electrodes resulted in a good desynchronization for excitatory stimulation, indicated by a vanishing synchronization measure R. In this case, (see Figure 9), the mean synchronization measure was R(t) = 0.15 for excitatory stimulation whereas without stimulation the index of synchronization was R(t) = 1.

These results, compared with those ones reported in the previous section, allow us to appreciate the same dynamical effect of the stimulation signals also in this reduced order network. In summary we used:

• Different, reduced order model consisting of *Izhikevich- Chattering;*

• Number of neurons equal to 225;

• Random network.

With this different system we obtained good values of index synchronization. For this reason we decided to continue the study with two coupled populations to study the mutual interaction effects.

**Figure 10:**
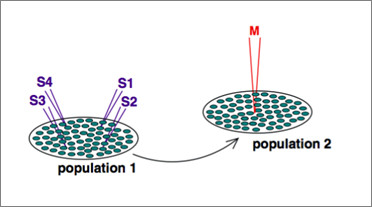
**Schematic plot of the stimulation setup**.

In this stimulation protocol we consider a setup, where a strongly synchronized neuronal population (population 1) acts as a pacemaker and drives another population (population 2), which gets synchronized only because of the driving. This structure resembles the case in which the pacemaker-like population in the BG and thalamus drives cortical motor areas which induce the peripheral shaking [22]. Consequently we modelled two neuronal populations (Figure 10): a driver (pacemaker) and a population (cortex) driven by the pacemaker via synaptic connections. Within each population the coupling is local, respectively, whereas the coupling strengths between the two populations are randomized and obey a Gaussian distribution.

To study the challenging situation of strong driving, we assume that the mean coupling within the driving population is equal to the mean coupling between the two populations. The excitatory coupling between the pacemaker and the driven system is given by . Within the driven population a weak excitatory synaptic coupling exists, which by itself does not induce synchronization. For illustration, we represent the neurons in the populations as arranged in square lattices and stimulated either via four stimulation electrodes equally spaced within the population or with one electrode situated in the central part of the network.

**Figure 11:**
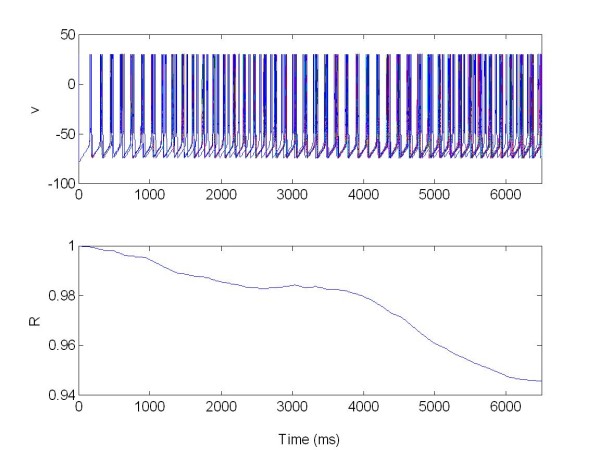
**The membrane potentials *v(t) *of the population 1 of 225 neurons connected with population 2 Figure 12**. The population was organized as a random network and not stimulated. R: Synchronization measure X = 0 at all times.

**Figure 12:**
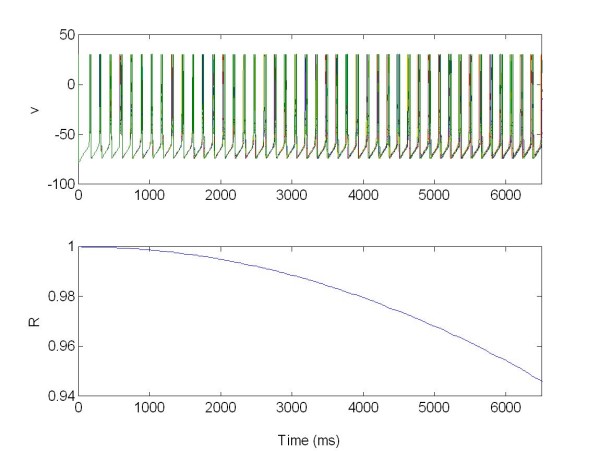
**The membrane potentials *v(t) *of the population 2 of 225 neurons connected to population 1 (see Figure11)**. The population was organized as a random network and not stimulated. R: Synchronization measure X = 0 at all times.

The local separation of stimulation and recording sites guarantees that the feedback signal is not corrupted by stimulation artifacts. Figure 11 and 12 show the two populations without stimulation but coupled together.

The stimulation in population 1 causes an instantaneous desynchronization of population 2, which shows a decrease of the synchronization measure R.

**Figure 13:**
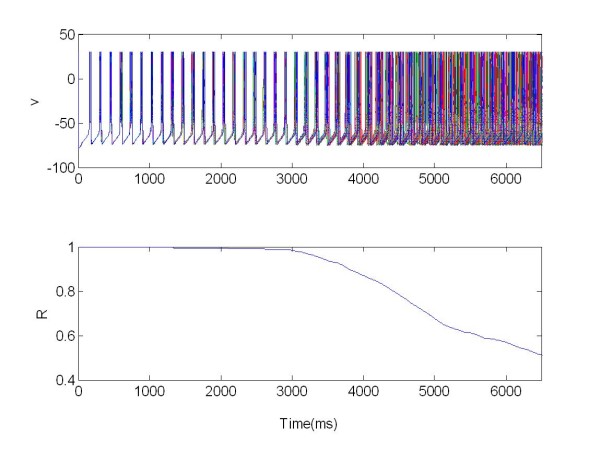
**The membrane potentials *v(t) *of the population 1 of 225 neurons connected with population 2 (see Figure 14)**. The population was organized as a random network and stimulated via one electrode. R: Synchronization measure X = 1 for t ∈ [3,6.5]s, excitatory stimulation; X = 0.

**Figure 14:**
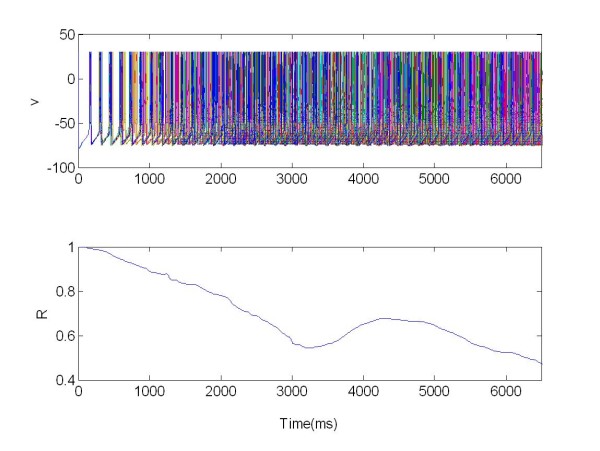
**The membrane potentials *v(t) *of the population 2 of 225 neurons connected with population 1 (see Figure 13)**. The population was organized as a random network and not stimulated. R: Synchronization measure.

In this case we use only one electrode to stimulate the coupled networks (Figure 13 and 14).

We can see that, when stimulated, the first population acts as peacemaker for population 2 (driven population), Figure 13, resulting in a decrease of the synchronization index R. For the first population R(t) = 0.7 (where t ∈ [3,6.5]s). For the second population, Figure 14, the value of R changes from an average of R(t) = 0.7 ∈ (when t 0[3]s), to R(t) = 0.5 (when t ∈ [3,6.5]s), as a consequence of the coupling with population 1.

**Figure 15:**
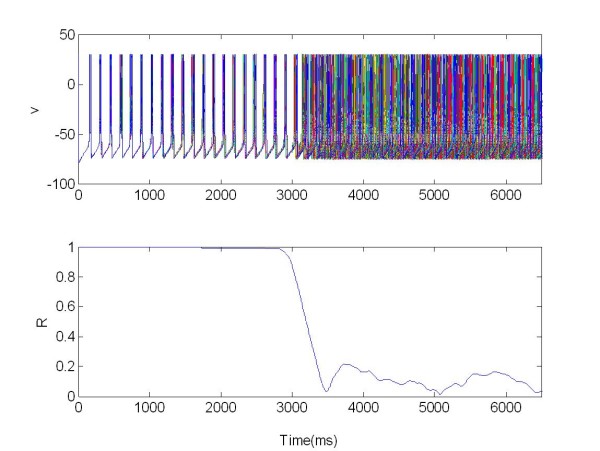
**The membrane potentials *v(t) *of the population 1 of 225 neurons connected with population 2, Figure 16**. The population was organized as a random network and stimulated via four electrodes. R: Synchronization measure X = 1 for t [3,6.5]s, excitatory stimulation; X = 0 elsewhere.

**Figure 16:**
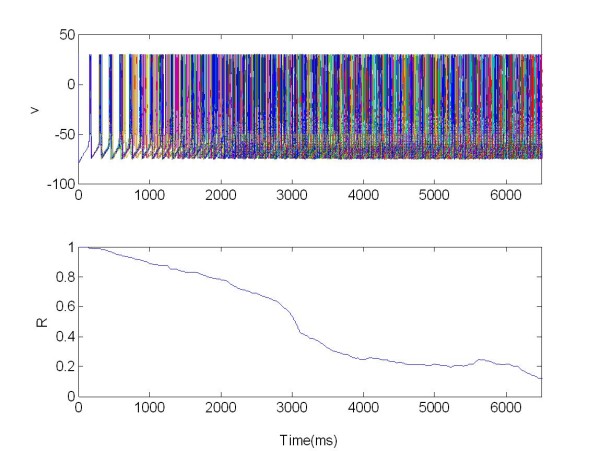
**The membrane potentials *v(t) *of the population 2 of 225 neurons connected with first population 1, Figure 15**. The population was organized as a random network and no stimulated. R: Synchronization measure.

The stimulation is realised via four electrodes located within the network. So we studied how the level of desyncronization of the driven population changes with a number of electrodes applied (Figure 15 and 16).

We can see in this simulation how the increasing the number of electrodes causes an increasing in the desynchronization effect. Stimulating the first population (Figure 15) acts as peacemaker for the driven population 2 (Figure 16).

For the first population we have R(t) = 0.2 where t ∈ [3,6.5]s, moreover for the second population the value of R change from R(t) = 0.5 with t ∈ 0[3]s, to R(t) = 0.3 with t ∈ [3,6.5]s, given by coupling with population 1.

## Discussion

The macroscopic effect of stimulation is represented by a modification of the Spectrograms with respect to the non-stimulated case. To appreciate the introduced model and its response to a stimulating pulse train, we calculate the LPF for our network model and translate the single burst activity into a macroscopic field measure. The LFP was calculated by an electrode positioned in the center of the neuronal population and was given by:(18)

where *r_j _*is the distance between neuron *j *and the recording electrode [50]. All the current components, except for the stimulation current, contributed to the ionic current *I_j_*(*t*)(Eq. 1). Therefore *I_j_*(*t*) is composed of , , ,  and the three ionic currents responsible for the basic spiking features of the *j_th _*STN model neurons (i.e., the right hand side of Eq. 1). R_e _= 1 is the extracellular resistivity that was assumed to be homogeneous.

**Figure 17:**
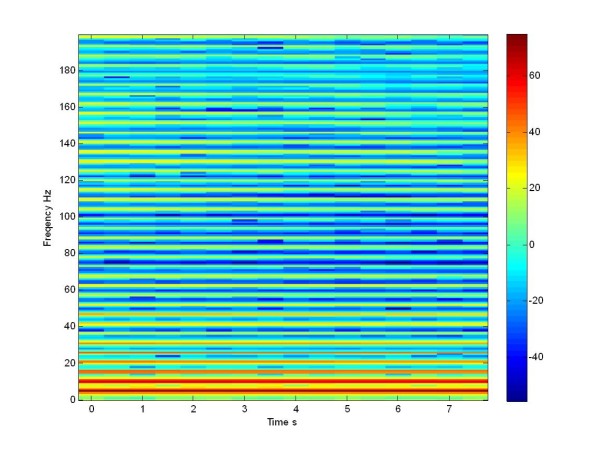
**Spectrogram in dB**. LFP of a not stimulated population of 100 Morris Lecar neurons.

The dynamics of the non stimulated network for the Moris Lecar model, in terms of LFP is reported (through the spectrogram in dB) in Figure 17. From this plot we can appreciate the presence of a high synchronization at low frequencies: this is the key characteristic of a pathological network, according to the neuronal gate theory. It is to be outlined that we conducted the study considering a very low number of neurons with respect to the actual case. Moreover relevant parameters, like the noise level and sources and the details of the network topology, in the in vivo case, were mostly unknown. Under this perspective, the results obtained can be considered relevant, above all in view of the effect of stimulation.

In the following the effect of stimulation is considered. We apply the traditional stimulation via four electrodes in the same 100 neuron population.

**Figure 18:**
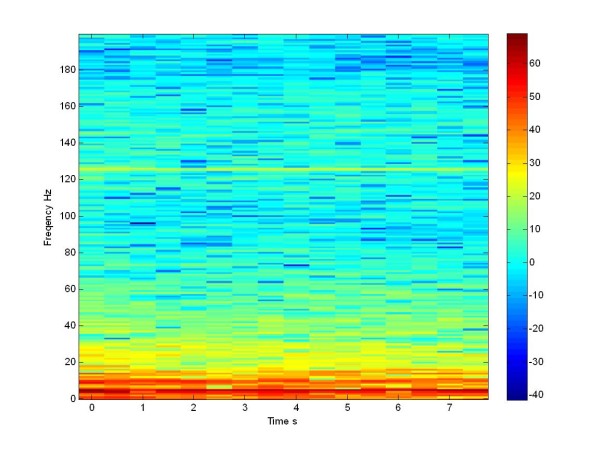
**Spectrogram in dB**. LFP of a stimulated population for all times with train pulse at 130 Hz of 100 Morris Lecar neurons.

When we apply the pulse train at 130 Hz the network desynchronizes particularly in the low frequency range, leading to a certain spreading of the spectral intensity, as we can see in Figure 18 for the Morris Lecar model. The results shown in Figure 18 can be compared with the literature [21] when the patient is treated with L-Dopa. These results were mostly important for our study: both in the un-stimulated and in the stimulated case, we can appreciate the same effect of stimulation, both in simulation and experimentally.

Referring to one population, in order to compare the dynamics of the un-stimulated and the stimulated network of the Izhijevich and Morris Lecar network, we studied how the application of a stimulation signal caused a change in the LFP of a population. As seen before, we will appreciate a desynchonization of the LFP through the spectrogram in dB of Izhikevich network. From Eq.18 we calculated the LFP of the Izhikevich population.

**Figure 19:**
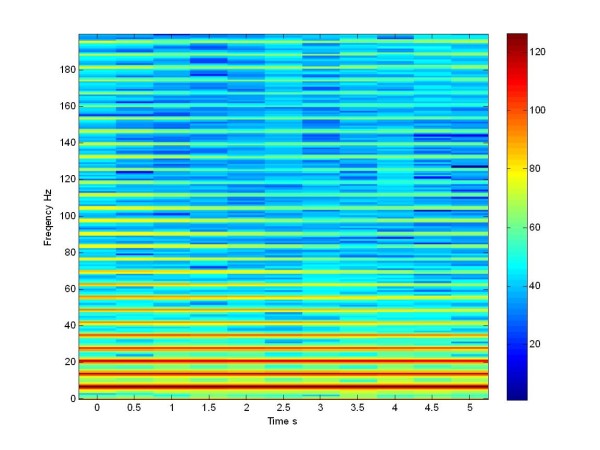
**Spectrogram in db**. LFP of a not stimulated population of 225 Izhikevich neurons.

Now we can compare this LFP simulated signal of Izhikevich neuron population with the LFP simulated signal of the Morris Lecar population (Figure 17, Figure 19), both not stimulated.

From these plots we can see that there is a high synchronization at low frequency and this is a characteristic, mimicking the pathological network. This behavior is clearly visible also in the Izhikevich neuron population: between the [5-20] Hz range there is a high activity.

**Figure 20:**
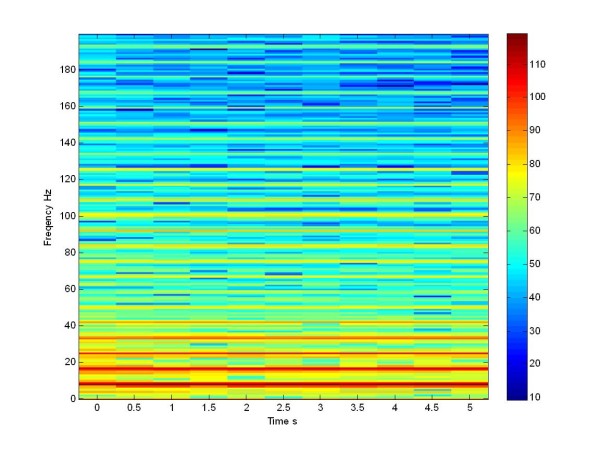
**Spectrogram in db**. LFP of a stimulated population for all times with train pulse at 130 Hz of 225 Izhikevich neurons High-frequency pulse train W(t) delivered through the electrode.

Subsequently we applied the traditional stimulation via four electrodes in a population (see Figure 20).

When we apply the pulse train at 130 Hz, the network desynchronizes at the low frequencies and we notice a spreading of the power density. The same effect is seen in the literature, when the patient is treated with L-Dopa [21], and in Figure 18, referring to the Morris Lecar network. If we compare the Figure 17 with Figure 19 and 18 with Figure 20, we can see that we obtained a same effects both in the stimulated and in the not stimulated case.

## Conclusions

In this work some models for studying the effect of stimulation in neural populations mimicking the dynamics met in the relevant brain neural tissues of PD patients are analysed. A new structure, built using a reduced order neuron model (the Izhikevich one), computationally much lighter, was demonstrated to lead to the same results as other neural networks already introduced in the literature. This gives the opportunity to more efficiently study the effect of stimulation in large scale neural networks. Results were compared with the other mathematical models, i.e. Morris Lecar neurons, analyzing both parameters related to the neural level (the synchronization effect) and indexes related to the macroscopic level (LFP). The results agree in terms of the positive effect of the stimulation in terms of power spectral density. We consider these results very promising: they open the way to the possibility of simulating large scale networks in pathological conditions at the aim to design new control strategies for the PD effect mitigation.

## List of abbreviations

**PD**: Parkinson's Disease; **DBS**: Deep Brain Stimulation; **BG**: Basal Ganglia; **FDA**: Food and Drug Administration; **STN**: Sub-Thalamic Nucleus; **GPi**: Globus Pallidus internus; **PPTg**: Nucleus Tegmenti Peduncolopontini; **PSD**: Power Spectral Density; **LFP**: Local Field Potential; **GPe**: Globus Pallidus externus; **VIM**: Ventral Intermediate Nucleus; **CM-Pf **Centrum Medianum-Parafascicular.

## Competing interests

The authors declare that they have no competing interests.

## Authors' contributions

AL, as the first author, designed and realized the simulation tool, carried out the simulations, refined the models and analysed the results; PA contributed in designing of the neural networks architectures and in revising the obtained results, further improving the neural models used; PM is the Nerurosurgeon who performed the surgical stereotactic procedures and contributed in revising the paper contents, according to his experience in the Deep Brain Stimulation field.

All authors read and approved the final manuscript.
